# Unforeseen Outcomes Post Treatment for Radiation Induced Trismus: A Case Report

**DOI:** 10.3390/medicines9050031

**Published:** 2022-04-19

**Authors:** Akash Sivam, Ankit Garg, Paul Sillifant

**Affiliations:** Oral and Maxillofacial Surgery Department, Royal Hobart Hospital, Hobart, TAS 7000, Australia; ankit.garg@ths.tas.gov.au (A.G.); paul.sillifant@ths.tas.gov.au (P.S.)

**Keywords:** radiation trismus, scar band release surgery, temporalis muscle flap, osteoradionecrosis, caries

## Abstract

Post radiotherapy radiation trismus presents significant concerns for a patient’s quality of life and for the clinical monitoring for recurrence of head and neck oncology. Current treatments include scar band release surgery that has been shown to be safe and effective. We present a case with a rare, post-operative complication of difficulty of mouth closure that can pose a significant impact on quality of life that should be considered.

## 1. Introduction

Radiotherapy plays an important role in the treatment of head and neck cancer. It can be indicated as a sole treatment modality or in combination with surgery and/or chemotherapy. Despite recent advances there remains a high risk of acute and chronic side effects with 50% of patients experiencing loco-regional side-effects [1]. Common side-effects include xerostomia, dental caries, periodontal disease, mucositis, skin changes and fibrosis. More debilitating side-effects are osteoradionecrosis (ORN) and post radiation trismus [2,3].

Post radiotherapy radiation trismus due to the fibrosis of the mandibular elevator muscles poses significant issues in the care of head and neck oncology patients [4]. It affects the clinician’s ability to monitor for recurrence and can reduced patient’s quality of life by affecting their oral intake and speech [5]. It is, therefore, prudent to monitor and treat patients’ radiation trismus. Current treatment options include both non-surgical and surgical procedures to release the scar band [6]. The case presented utilises a surgical scar band release with the use a temporalis muscle flap and split thickness skin graft to reconstruct the defect. These procedures are well tolerated and have been shown to be safe with few adverse outcomes [7,8,9]. This is the only case in the current literature that highlights a rare complication of difficulty of mouth-closing and subsequent post-operative difficulty with speech and swallowing as well as with the patient’s rehabilitation.

## 2. Case Report

A 57-year-old female, with known T2N1M0 right tonsillar squamous cell carcinoma, who had undergone wide local excision with ipsilateral selective neck dissection with subsequent chemoradiotherapy (68 Gy in 34 fractions), was referred for worsening trismus and myofascial pain over six years that had not improved with botulin toxin injections, bilateral coronoid process removals and manipulations under anaesthesia, which were performed by another surgical unit. When she presented to our unit she described significant limitation in mouth opening, with subsequent limitations in speech, oral intake and difficulty maintaining oral hygiene. Her past medical history also included osteoradionecrosis (ORN) of the jaw. Examination was limited due to trismus but her interincisal opening was 2 mm with resulting poor oral hygiene.

**Figure 1:**
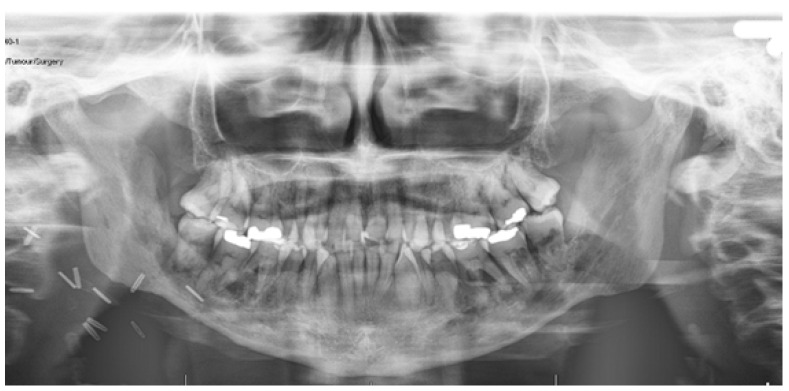
Pre-operative OPG.

**Figure 2:**
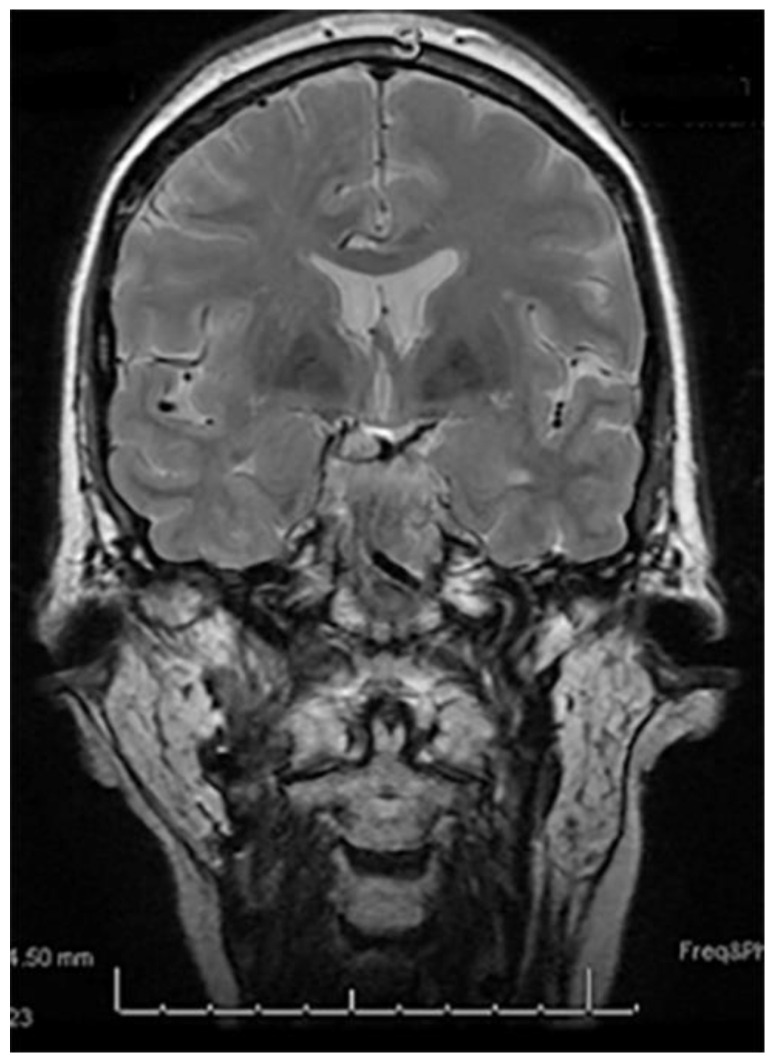
Pre-operative MRI.

Orthopantomogram (OPG) and computed tomography (CT) demonstrated removal or left coronoid, right coronoid in situ and bilateral mandible ORN (Figure 1). Magnetic resonance imaging (MRI) showed degeneration of the right articular disc and limited anterior translation of mandibular condyles with respect to mandibular fossa (Figure 2).

An intra-oral scar band release, bilateral temporalis flap, split thickness skin graft (STSG), bilateral posterior dental clearance and mandible corticotomy and tracheostomy was performed after completing 34 treatments of hyperbaric oxygen therapy (HBOT) under general anaesthesia. An awake nasal fibreoptic intubation was performed due trismus, 35 mL of 0.5% Marcaine + 1:100,000 adrenaline was administered intra-orally as local anaesthesia. Dental extractions of all posterior molars with alveoloplasty and corticotomy of ORN bone until bleeding was performed. Bilateral mandibular vestibular incisions of 40 mm were made, with blunt dissection exposing the mandibular ramus and posterior bodies. The right coronoid process and bilateral scar tissue from masseter and medial pterygoid was excised, leaving defects approximately 25 × 35 mm in size. Bilateral hemicoronal incisions extending inferiorly to the preauricular region at the level of the tragus were made to harvest the temporalis muscle flaps. Careful dissection was carried to the posterior border of the temporalis muscles with care to avoid frontal branch of the facial nerve. The posterior temporalis muscles were detached, and the temporalis muscles were elevated and mobilised. Dissection continued under the zygomatic arches allowing for rotation of the temporalis muscles and approximation into the scar cavity where they were secured with simple interrupted 3–0 chromic gut. The scar cavity was reconstructed with a temporalis muscle flap and a split thickness skin graft, harvested from left thigh. Size 10 Blake drains were placed in the preauricular incisions and secured with 2–0 silk. Surgical wounds were closed with simple interrupted sutures using 3–0 chromic gut (intra-oral) and 4–0 prolene, as well as staples (extra-oral). A surgical tracheostomy was performed and secured with 2–0 silk, and a nasoenteric tube and bite block were inserted at the end of the procedure. Other than minor intraoperative bleeding that was controlled with tamponade and cautery, no intraoperative complications were encountered during the surgery.

Post-operatively the patient was initially managed in ICU and underwent eight treatments of HBOT. On day 1 post operation, Blake drains were removed as output was less than 30 mL over 24 h, her bite block was removed, and she was noted to have mouth opening greater than 35 mm that progressed to 40 mm by day 3. On day 5 post operation, weak mouth opening was achieved; this was helped with gentle chin pressure, and the patient was instructed to perform jaw closing and swallowing exercises for which she had ongoing physiotherapy and speech pathology support during her admission. The patient was eventually discharged to rehabilitate with ongoing speech pathology input, where she has had a percutaneous endoscopic gastrostomy (PEG) tube inserted due to poor swallowing.

**Figure 3:**
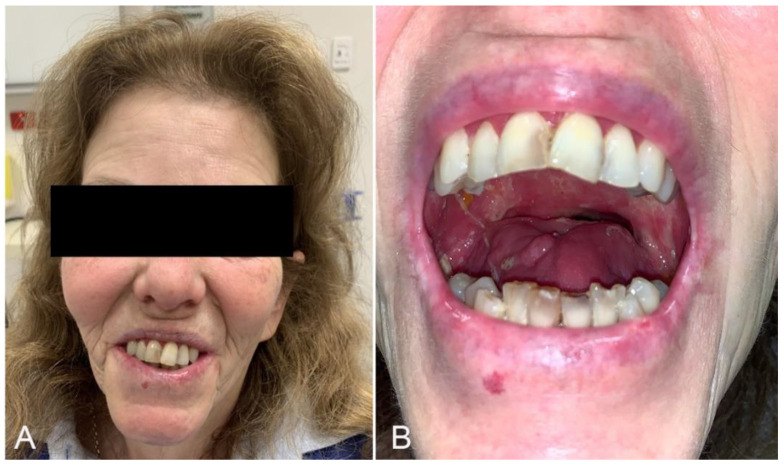
(**A**) 8 month post-operative unsupported mouth closure; (**B**) 8 month post-operative mouth opening.

The patient had ongoing intensive speech pathology input and was reviewed post-operatively after 2 months, where her mouth opening was 40 mm and wounds had healed, however, she had difficulty swallowing and was unable to contact incisors together without chin support. In her 4 month post-operative review she displayed noted improvement in mouth closing with mandibular closing power 4/5 (Medical Research Council’s scale MRC); however, the patient retained ongoing difficulty swallowing, requiring chin support to swallow. Eight months post operation she had a mouth opening of 40 mm and good unsupported mouth closure (Figure 3A,B), facilitating her ability to swallow a minced diet. As a result, her PEG tube was removed, and she was discharged from rehabilitation.

## 3. Discussion

Radiotherapy, along with surgery and chemotherapy, is a major treatment modality for head and neck cancers [10]. While radiotherapy provides successful results in increasing survival rates of head and neck cancer patients and improved quality of life (QoL), complications such as dry mouth, oral mucositis, loss of taste, caries, trismus, and osteoradionecrosis (ORN) are encountered [4,11].

Oral mucositis is an acute response to radiotherapy for head and neck cancer [12]. Oral mucositis peaks near the end of radiotherapy treatment and continues for 2–4 weeks post treatment, with recovery taking place over several weeks [13]. It commonly presents with erythema, ulcerations, and severe pain. As a result of pain, patients note pain related trismus and a decrease in oral intake with subsequent weight loss, poor nutrition, and poor healing [14].

Osteoradionecrosis of the jaw is a late complication of radiotherapy, affecting approximately 4–8% of patients [15]. Symptoms include pain, trismus, necrotic bone, oronasal fistulas or pathological fractures [16]. Severe trismus has been shown to effect 7.1% of oncological patients with ORN, thereby causing malnutrition and cachexia with negative impacts on QoL [17]. Median time to develop ORN is 18 months post radiotherapy with risk factors including poor dental status prior to radiotherapy, radiation dose of Dmax >60 Gy, planning target volume of >40% of the jaw and dental extractions or trauma in the radiation field (89.4%) [18,19,20]. Curi and Dib demonstrated with conservative treatment of antibiotics and optimization of oral hygiene that 42.3% of cases had complete healing and resolution, 32.6% cases had stable or chronic ORN and 25.1% had acute and progressive ORN [19].

Normal maximal mouth opening is 40–45 mm (interincisal). Radiation trismus is defined as a reduction in mouth opening of less than 35 mm, resulting from inflammation and subsequent fibrosis of the mandibular elevator muscles due to the ionising radiation effects of radiotherapy [21].

A systematic review investigating trismus in head and neck cancer patients found the prevalence to be 17% at baseline, 44% at 6 months post radiotherapy, 32% at 12 months and 3–10 years post radiotherapy [22]. Further studies reveal that 50% of patients can have a limitation of the temporomandibular joint (TMJ) and of masticatory muscles activity post radiotherapy [23]. Trismus is dependent on radiation dose, site and number of fields radiated. It can progress over the course of months and early intervention, such as mouth opening exercises, is recommended to prevent trismus [24].

Trismus complicates post cancer care by impeding the clinician’s ability to examine for recurrence, and the patient’s QoL by negatively affecting their ability to eat, drink and talk normally, and form dental prosthesis [5,10]. The management of trismus can include forced mandible opening, use of opening devices and modification prosthesis and surgery to release scar band [6].

Rai et al. investigated intra-oral scar tissue release, finding an improvement in mouth opening, and complications of flap necrosis, temporary widening of oral commissure and subluxation of TMJ. They recommended monitoring for recurrence of strictures post-operatively [7]. In our case, temporalis muscle flaps and STSG were used in conjunction with scar band release to aid in mouth opening. A temporalis muscle (TM) flap is a versatile tool in head and neck surgery. It can be used as a reconstructive option to reconstruct various defects. TM flaps are well vascularised with low rates of necrosis, large enough to fill large defects and provide good outcomes for speech and swallowing [9].

Functionally, Browne et al. [25] assessed the post-operative function of 27 patients with TM flaps and found that the mean nasalance (defined as the degree of velopharyngeal opening in voiced speech) were within the normal limits for connected speech tasks. Furthermore, swallowing scores in this study measured via MDADI (MD Anderson Dysphagia Inventory score) ranged from good to mildly affected [25]. Additionally, both Brennan et al. and Ahmed et al. commented that the post-operative function of speech and swallowing was good and unaffected [8,9]. Moreover, when investigating the use of TM flaps for TMJ disorders, studies have found improvement in pain, increased mouth opening of 11.1 mm and lateral excursion for up to 3 months, without any major complications [26,27].

The present case represents a complication not encountered in the current literature. There are no previous reports of scar band release and reconstruction with TM flaps resulting in inability to close the mouth and a subsequent difficulty in swallowing. In this case it is postulated that severe trismus was due to a combined effect of post radiation mucositis, ORN and a prolonged non-use of masticatory muscles with previous radiotherapy rendering these muscles atrophic and fibrosed. Post surgery, these weakened muscles, in particular the intact masseter and medial pterygoid muscles, lacked the power required to close the patient’s mouth. As a result, our patient was unable to produce enough increase in intra-oral pressure to facilitate the transport of food to the oropharynx or raise the base of the tongue to the posterior pharyngeal wall, thus initiating a pharyngeal squeeze necessary for swallow [28]. Successful treatment of this patient relied on a multidisciplinary team approach. Radiation oncologists and primary care physicians provided symptomatic relief to aid in reduction of pain and, therefore, trismus. Dentists ensured maintenance of oral hygiene in a difficult case to prevent further complications. Hyperbaric oxygen physicians administered hyperbaric oxygen treatment for ORN. Oral and maxillofacial surgeons performed the surgery detailed to treat ORN, release scar tissue and improve trismus. Speech pathologists provided masticatory muscle exercises and appliances to strengthen these muscles, improving mouth closure and, therefore, oral intake and nutrition. Rehabilitation physicians ensured the patient received adequate nutrition while oral intake was compromised.

## 4. Conclusions

This case acts as a cautionary tale to highlight the effects of muscle weakness post prolonged periods of non-use, and their effects on post-surgery treatment of trismus, i.e., the inability to close the mouth and swallowing difficulties. This case also identifies the importance of a multidisciplinary team including allied health input, such as speech pathology and rehabilitation for rehabilitating patients to improve function post-operatively. We hope that by highlighting this, clinicians are better placed to inform patients of the expected complications and their treatment and progression.
